# The comorbidity of anxiety and depression symptoms in obsessive–compulsive disorder: a network analysis

**DOI:** 10.3389/fpsyt.2025.1567448

**Published:** 2025-05-02

**Authors:** Sizhe Cheng, Zhiguo Chen, Chao Wu, Xinhong Li, Xin Shen, Rui Qiu, Nailong Tang, Chong Feng, Wenwen Wang, Jing Lv, Shuiping Yuan, Xufeng Liu

**Affiliations:** ^1^ School of Military Medical Psychology, Air Force Medical University, Xian, China; ^2^ Department of Psychiatry, No.907 Hospital of Joint Logistics Support Force of People’s Liberation Army (PLA), Nanping, China; ^3^ School of Nursing, Air Force Medical University, Xian, China; ^4^ Department of General Medicine, Tangdu Hospital, Air Force Medical University, Xi’an, China; ^5^ Department of Health Statistics, School of Preventive Medicine, Ministry of Education Key Lab of Hazard Assessment and Control in Special Operational Environment, Air Force Medical University, Xian, China; ^6^ The Second Medical Center & National Clinical Research Center for Geriatric Diseases, Chinese People’s Liberation Army (PLA) General Hospital, Beijing, China

**Keywords:** obsessive-compulsive disorder, anxiety, depression, network analysis, comorbidity

## Abstract

**Objective:**

People with obsessive–compulsive disorder (OCD) frequently suffer from comorbid anxiety and depression. From the perspective of the network model, this comorbidity is thought to be an interacting system of three symptoms. In the study, we conducted a network analysis of anxiety and depression comorbidity in OCD, aiming to identify the central and bridge symptoms and make informed suggestions for clinical interventions and psychotherapy.

**Methods:**

A total of 356 individuals with OCD were enrolled in the study. The Yale–Brown Obsessive–Compulsive Scale (Y-BOCS), Self-Rating Anxiety Scale (SAS), and Self-Rating Depression Scale (SDS) were selected to evaluate anxiety, depression, and obsessive–compulsive symptoms, respectively, followed by network analysis to construct the interacting networks.

**Results:**

“Panic” and “Distress caused by obsessive–compulsive behavior” had the highest expected influence, indicating that the two variables were the most associated nodes in the network. “Tachycardia,” “Constipation,” “Fatigue,” “Panic,” “Easy fatiguability, weakness,” “Palpitation,” “Crying spells,” “Psychomotor agitation,” “Mental disintegration,” and “Nightmares” were the bridge nodes that had the strongest connection with OCD.

**Conclusions:**

Panic and distress caused by obsessive-compulsive, which are the central and bridge symptoms of the interacting network of anxiety and depression symptoms in OCD, might be a significant transdiagnostic intervention target for the management of the comorbidity. Additionally, it might be beneficial to consider implementing clinical prevention and psychotherapy, focusing on somatic symptoms, psychological symptoms, and sleep that have the strongest associations with OCD.

## 1 Introduction

Obsessive–compulsive disorder (OCD) is a mental disorder characterized by the presence of ego-dystonic intrusive thoughts (obsessions) and mental or physical rituals (compulsions), which take up a considerable amount of time (1 h or more per day) or cause significant distress or impairment (1). OCD affects up to 3% of the population (2), associated with a low quality of life, personal and social burden, loss of functioning and productivity, and high levels of healthcare service use (3–5). Notably, OCD is often present with comorbid conditions. The prevalence of OCD with psychiatric comorbidity can reach approximately 62% (6) and even up to 79.7% (7), which reflects the phenomenon that comorbidity is the norm and not the exception when it comes to OCD.

Anxiety and depression are the most common manifestations of psychological abnormalities, which may occur in everyone’s lifetime (8). Both OCD and anxiety disorders belong to the category of neurosis, and the latter had been included in the definition of the former according to the *Diagnostic and Statistical Manual of Mental Disorders, Fourth Edition* (*DSM-IV*) (9), which have a clinical sign of common etiopathogenetic factors, including a common genetic and neurobiological background, expressed in features such as the domination of negative affect, neuroticism, avoidance of threats, or intolerance of uncertainty (10–13). Moreover, research on twins indicates a common genetic background for OCD and anxiety disorders, suggesting that obsessive–compulsive (OC) symptoms may be a risk factor for the genesis and development of some anxiety disorders, such as generalized anxiety disorder (GAD) and panic disorder (PD) (14, 15). Major depression is considered the most common comorbidity of OCD with lifetime rates estimated at 62.7%–78.2% (16, 17), accompanied by increased risk of suicide attempts (18, 19), functional disability (20), and elevated OCD symptom severity (21). Although comorbid anxiety and depression are both clinically common and worrisome, the mechanisms of comorbidity are poorly understood.

Recently, the data-driven network model, which can analyze and intuitively display the relationships among multiple variables, has been utilized to conceptualize mental disorders by fitting data with a Gaussian image model (22). It is based on the mathematical analysis and visual representation of the interaction between complex variables, which has the advantage of not having to rely on previous assumptions about the relationship among variables and allowing visualization of the association patterns of different variables (23). The network usually consists of nodes and edges, which construct communities. Nodes refer to the variable, or rather, the symptoms of psychological disorders, while edges refer to the connections among the variables (24). Community refers to a group of psychological variables. The bridge centrality index helps to accurately capture variables that play an important role in connecting communities in the whole network (25, 26). From a network point of view, mental disorders are not single underlying latent variables but the direct interactions among psychiatric symptoms (27). Network analysis can build a structural network, reflecting not only the weight item of psychological scale, but also the complex interrelationship among different items. The network can identify central symptoms that determine the severity of mental disorders and deserve the utmost clinical attention and targeted attention. Additionally, bridge symptoms, which play a vital role in the risk of other disorders, also provide new insights into the occurrence of comorbidities and inspirations for intervention (28).

In this study, we explored the relationship among OCD symptoms, anxiety, and depression in OCD using network analysis. We constructed the network model and estimated the bridge centrality to determine the important role of specific aspects of the OCD’s comorbidity with anxiety and depression and identified the variables connecting these three communities to provide perspective on targeted intervention.

## 2 Methods

### 2.1 Participants

A total of 356 individuals with OCD, followed up at the Department of Psychiatry or Medical Psychology, No. 907 Hospital of Joint Logistics Support Force of PLA (Nanping, China), Chinese PLA Hospital (Beijing, China), and Tangdu Hospital (Xian, Chian), were recruited in the study from December 2022 to June 2024. This study was approved by the Ethics Committee of Xijing Hospital, Air Force Medical University (KY20243604-1). All participants or their legal guardians consented to the study according to the Declaration of Helsinki.

### 2.2 Evaluating OCD, anxiety, and depression symptoms

The Yale–Brown Obsessive–Compulsive Scale (Y-BOCS), Self-Rating Anxiety Scale (SAS), and Self-Rating Depression Scale (SDS) used in the study are the quantitative evaluation criteria recommended by *DSM-V*. The questionnaires have simple content and strong maneuverability, which have been proven to have good reliability and validity (29, 30).

Y-BOCS is a 13-item manualized, semi-structured interview to measure the severity of OCS over the past week (31). It is composed of a comprehensive symptom checklist to identify the specific type and content of OCD symptoms, which is considered to be the gold standard measure of OCD. The preliminary item evaluates the occurrence of OC symptoms. When OC symptoms are present, the interview continues with five items that assess the severity of obsessions and compulsions, respectively. All items are rated on a five-point Likert scale, ranging from 0 (no symptom) to 4 (extreme symptoms). The originally proposed scoring structure consists of three summary scores: (1) Obsession (range = 0–20) is the sum of the five items that evaluate the severity of obsessions. (2) Compulsion (range = 0–20) is the sum of the five items that examine the severity of compulsions. (3) The total score (range = 0–40) is the sum of all 10 items. A total score of 10–20, 21–30, and 31–40 indicates mild, moderate, and severe OC symptoms, respectively. In previous studies, the Y-BOCS (Chinese version) has been widely used in psychological evaluation related to clinical treatment effects and other aspects (32, 33).

The SAS is a simple and efficient self-assessment tool for evaluating anxiety symptoms, which is widely used in the psychiatric and psychological clinic (34). The scale has 20 self-rate items that are scored on a four-point Likert scale according to the frequency of symptoms in the past 7 days, ranging from 1 to 4. The standard score is equal to the raw score multiplied by 1.25, which is calculated by the sum of each item. The cutoffs for the SAS standard scores are defined as follows: no anxiety (<50), minimal to mild anxiety (50–59), moderate to marked anxiety (60–69), and severe anxiety (≥70) (35). The SAS (Chinese version) has been widely used and demonstrated to have adequate reliability and validity (35, 36).

The SDS is a 20-item reliable self-rating scale designed for screening the mood symptoms in the past 7 days (37). Each item is scored on a Likert scale ranging from 1 to 4 according to the frequency over the past week. The standard score is equal to the raw score multiplied by 1.25, which is calculated by the sum of each item. Standard SDS scores are classified as follows: no depression (<50), minimal to mild depression (50–59), moderate to marked depression (60–69), and severe depression (≥70). The SDS (Chinese version) has been confirmed in previous studies (38, 39).

### 2.3 Network analysis

#### 2.3.1 Network estimation and visualization

Gaussian graphical models (GGMs) were employed to assess the structural relationships within data via the R package *qgraph* for network estimation (40). GGMs facilitate the construction of a non-oriented network where each edge denotes the partial correlation between a pair of nodes, adjusting for the influence of all other nodes in the system (41). The GGMs’ estimation was based on nonparametric Spearman’s rho correlation matrices, providing a robust framework for analyzing the relationships between variables. To refine the network, the graphical least absolute shrinkage and selection operator (LASSO) algorithm was utilized for regularization, by reducing the impact of less significant edges and eliminating those with minimal partial correlations, thereby yielding a simplified non-oriented weighted network (42). The tuning parameter was set to 0.5 to strike a balance between sensitivity and specificity for enhancing edge detection (43). Network visualization was facilitated by the Fruchterman–Reingold algorithm, which located the nodes with stronger correlations near the center of the network and weaker correlations on the periphery (44). Within the network, edges are color-coded to denote correlations: green for positive and red for negative partial correlations, with edge thickness denoting the strength of the correlation between nodes.

In order to further correct the influence of gender and age, which are potential confounding factors, on the results of the network analysis, we conducted stratified sub-network analyses by gender (designated as “male” and “female”) and age (divided into those under 18 years, those who are 19–30 years old, and those over 30 years) among the participants. Subgroup networks were estimated using identical regularization parameters and bootstrapping procedures as the primary analysis. The results are shown in **Supplementary Figures S1** and **S2**.

#### 2.3.2 Expected influence and predictability analysis

The node expected influence within the network is calculated via the R package *qgrph* (45). Nodes with higher expected influence values are considered more pivotal within the network structure. Furthermore, the R package *networktools* was utilized to compute the bridge expected influence (BEI), which quantified the connectivity between nodes in different communities (46). A higher BEI indicates a node’s potential to influence other communities (47). Bridge symptoms were identified using a data-driven threshold derived from bootstrapped BEI scores. Specifically, we performed 2,000 bootstrap resamples to generate a distribution of BEI values for each node. The 95% confidence interval (CI) of the BEI distribution was calculated, and nodes whose mean BEI exceeded the upper bound of the 95% CI (equivalent to the 97.5th percentile) were classified as bridge symptoms. This approach ensures that only nodes with statistically robust bridge centrality are retained, reducing reliance on arbitrary thresholds (46). Additionally, the predictability of the nodes was calculated via the R package *mgm*, which measures how well the variance of a node is accounted for by its connected nodes, thus reflecting the node’s controllability within the network (48). Nodes with high predictability are more easily managed through their neighboring nodes, whereas those with low predictability may require direct intervention or consideration of external variables.

#### 2.3.3 Network accuracy and stability

The R package *bootnet* algorithm was utilized to assess the stability of the network (49). First and foremost, the stability of the edges in the network is examined through a resampling procedure, where the observed values were replaced with bootstrapped estimates (*nboots* = 2,000) to establish a 95% CI for each edge. Edges that demonstrated minimal overlap with the 95% CI were considered essential for network stability. Subsequently, the case-dropping bootstrap method (*nboots* = 2,000) was employed to calculate the correlation stability coefficient (CS), which assesses the robustness of expected influences and BEIs. The CS indicates the maximum number of nodes that can be removed while maintaining a correlation with the initial centrality index above the threshold of 0.7. A CS value above 0.5 signifies robust node stability, with the minimum acceptable CS being 0.25 (49). Finally, bootstrapped difference tests (*nboots* = 2,000, α = 0.05) were employed to detect significant differences in edge weights, expected influences of nodes, and BEIs.

## 3 Results

### 3.1 Basic descriptive characteristics of anxiety, depression, and OC symptoms

A total of 356 individuals with OCD (73 women and 283 men) were recruited in the study. The mean age was 23.29 ± 7.19 years (mean ± SD, range 13–60 years). The Y-BOCS reflects the severity of OCD, while SAS and SDS reflect the severity of comorbidity with anxiety and depression symptoms. Detailed demographic characteristics and severity of OCD, anxiety, and depression symptoms are listed in **Tables 1** and **2**, respectively. **Table 3** shows the means, SDs, and predictability for each item of the Y-BOCS, SAS, and SDS.

**Table 1:** Detailed demographic characteristics of 356 patients with OCD.

Variables	Mean	SD
Age	23.29	7.19
	Number	Percentage (%)
**Gender**		
Male	283	79.49
Female	73	20.51
**Education**		
Junior high school and below	49	13.76
Senior high school or technical secondary school	76	21.35
Junior college	152	42.70
Undergraduate	74	20.79
Master	4	1.12
Doctor	1	0.28
**Marital status**		
Married	47	13.20
Unmarried	304	85.39
Divorced	4	1.12
Be bereaved of one’s spouse	1	0.28
**Registered permanent residence**		
Urban area	149	41.85
Rural area	207	58.15

**Table 2:** Severity of OCD, anxiety, and depression symptoms.

Variables	Number	Percentage (%)
OCD symptoms		
No to minimal symptoms (0–9)	91	25.56
Mild symptoms (10–20)	133	37.36
Moderate symptoms (21–30)	110	30.90
Severe symptoms (31–40)	22	6.18
Anxiety symptoms		
No anxiety (<50)	94	26.40
Minimal to mild anxiety (50–59)	85	23.88
Moderate to marked anxiety (60–69)	80	22.47
Severe anxiety (≥70)	97	27.25
Depression symptoms		
No depression (<50)	48	13.48
Minimal to mild depression (50–59)	47	13.20
Moderate to marked depression (60–69)	64	17.98
Severe depression (≥70)	197	55.34

**Table 3:** Mean scores, SD, and predictability for each symptom of the Y-BOCS, SAS, and SDS.

Symptoms	Mean	SD	Predictability
**OCD symptoms**	17.00	9.10	
Y-BOCS-1: Time and frequency of OC thinking	2.97	1.03	0.74
Y-BOCS-2: Social contact or work affected by OC thinking	2.71	1.14	0.54
Y-BOCS-3: Distress caused by OC thinking	3.04	1.16	0.69
Y-BOCS-4: Resistance to OC thinking	2.55	1.26	0.72
Y-BOCS-5: Control of OC thinking	2.77	1.32	0.67
Y-BOCS-6: Time and frequency of OC behavior	2.59	1.07	0.58
Y-BOCS-7: Social contact or work affected by OC behavior	2.37	1.35	0.54
Y-BOCS-8: Distress caused by OC behavior	2.48	1.42	0.54
Y-BOCS-9: Resistance to OC behavior	2.36	1.47	0.74
Y-BOCS-10: Control of OC behavior	2.54	1.40	0.58
**Anxiety symptoms**	59.59	15.22	
SAS-1: Anxiousness	2.71	0.93	0.70
SAS-2: Fear	2.44	0.96	0.64
SAS-3: Panic	2.79	0.88	0.59
SAS-4: Mental disintegration	2.29	1.02	0.74
SAS-5: Apprehension	1.94	0.92	0.81
SAS-6: Tremors	1.89	0.87	0.66
SAS-7: Body aches and pains	2.32	1.03	0.73
SAS-8: Easy fatiguability and weakness	2.90	0.89	0.68
SAS-9: Restlessness	2.12	0.91	0.83
SAS-10: Palpitation	2.13	0.89	0.65
SAS-11: Dizziness	2.16	1.00	0.61
SAS-12: Faintness	1.83	0.91	0.74
SAS-13: Dyspnea	2.61	1.00	0.98
SAS-14: Paresthesia	1.72	0.89	0.73
SAS-15: Nausea and vomiting	2.01	1.00	0.89
SAS-16: Urinary frequency	2.12	0.90	0.82
SAS-17: Sweating	2.19	1.02	0.95
SAS-18: Face flushing	1.73	0.83	0.89
SAS-19: Insomnia	1.76	0.92	0.66
SAS-20: Nightmares	2.31	0.97	0.78
**Depression symptoms**	69.25	16.26	
SDS-1: Depressed affect	3.08	0.84	0.74
SDS-2: Diurnal variation	1.61	0.84	0.86
SDS-3: Crying spells	2.42	0.98	0.73
SDS-4: Sleep disturbance	2.96	0.97	0.63
SDS-5: Decreased appetite	2.16	1.04	0.88
SDS-6: Decreased libido	1.78	1.02	0.81
SDS-7: Weight loss	1.98	1.01	0.92
SDS-8: Constipation	1.85	0.97	0.92
SDS-9: Tachycardia	2.13	0.96	0.63
SDS-10: Fatigue	2.89	0.95	0.68
SDS-11: Confusion	2.19	1.01	0.76
SDS-12: Psychomotor retardation	2.16	1.02	0.78
SDS-13: Psychomotor agitation	2.66	0.98	0.64
SDS-14: Hopelessness	1.97	1.03	0.69
SDS-15: Irritability	2.47	1.00	0.86
SDS-16: Indecisiveness	1.77	0.90	0.76
SDS-17: Personal devaluation	1.87	0.95	0.69
SDS-18: Emptiness	1.72	0.87	0.63
SDS-19: Suicidal rumination	2.27	1.08	0.72
SDS-20: Dissatisfaction	2.00	1.01	0.71

OC, obsessive–compulsive.

### 3.2 Network structure of OC symptoms, anxiety, and depression in individuals with OCD

**Figure 1:**
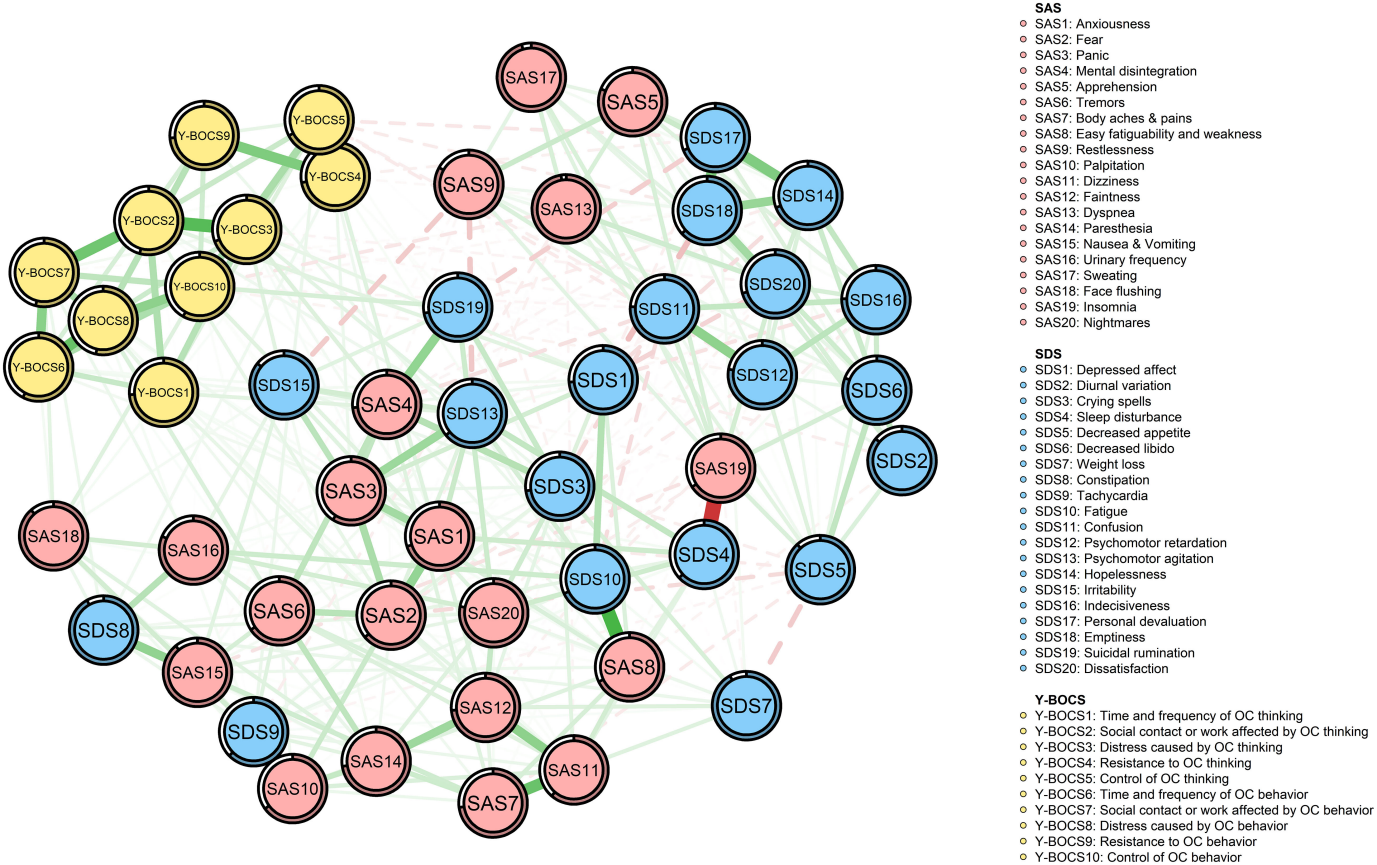
Network structure of OCD, anxiety, and depression symptoms. Green edges represent positive partial correlations; red edges represent negative partial correlations. Edge thickness corresponds to correlation strength. Node predictability is indicated by the surrounding circles. Nodes are color-coded by symptom domain: red (SAS), blue (SDS), and yellow (Y-BOCS).

The network model is shown in **Figure 1**. There were a total of 373 edges (range from −0.41 to 0.52) in the whole network and 187 edges (range from −0.41 to 0.52) across the OC symptoms, anxiety, and depression communities in the network (OC symptoms–depression: 39 edges; OC symptoms–anxiety: 36 edges; anxiety–depression: 112 edges).

In the edges across communities, “time and frequency of OC thinking” (YB1) was positively correlated with two nodes of the depression community (SDS1 and SDS13) and seven nodes of the anxiety community (SAS1, SAS2, SAS7, SAS8, SAS11, SAS12, and SAS18), among which the correlation with “Psychomotor agitation” (SDS13) and “Faintness” (SAS12) was the strongest, respectively (edge weight: 0.06 and 0.05).

Not only was “Social contact or work affected by OC thinking” (YB2) positively correlated with three nodes of the depression community (SDS1, SDS9, and SDS13) and two nodes of the anxiety community (SAS2 and SAS4), among which the correlation with “Depressed affect” (SDS1) and “Fear” (SAS2) was strongest, respectively (edge weight: 0.06 and 0.05), but it was also negatively correlated with two nodes of the depression community (SDS11 and SDS12), among which the correlation with “Confusion” (SDS11) was the strongest (edge weight: −0.02).

Not only was “Distress caused by OC thinking” (YB3) positively correlated with five nodes of the depression community (SDS1, SDS3, SDS13, SDS15, and SDS19) and three nodes of the anxiety community (SAS1, SAS4, and SAS12), among which the correlation with “Irritability” (SDS15) and “Mental disintegration” (SAS4) was stronger, respectively (edge weight: 0.06 and 0.07), but it was also negatively correlated with two nodes of the depression community (SDS6 and SDS20), among which the correlation with “Dissatisfaction” (SDS20) was the strongest (edge weight: −0.02).

“Resistance to OC thinking” (YB4) was not only positively correlated with three nodes of the depression community (SDS8, SDS10 and SDS19) and two nodes of the anxiety community (SAS3 and SAS12), among which the correlation with “Constipation” (SDS8) and “Mental disintegration” (SAS4) was stronger, respectively (edge weight: 0.03 and 0.06), but also negatively correlated with two nodes of the depression community (SDS17 and SDS18), among which the correlation with “Personal devaluation” (SDS17) was the strongest (edge weight: −0.03).

Not only was “Control of OC thinking” (YB5) positively correlated with three nodes of the depression community (SDS1, SDS3, and SDS9) and three nodes of the anxiety community (SAS4, SAS11, and SAS12), among which the correlation with “Tachycardia” (SDS9) and “Mental disintegration” (SAS4) was stronger, respectively (edge weight: 0.04 and 0.03), but it was also negatively correlated with four nodes of the depression community (SDS11, SDS12, SDS17, and SDS18), among which the correlation with “Personal devaluation” (SDS17) was the strongest (edge weight: −0.05).

“Time and frequency of OC behavior” (YB6) was positively correlated with three nodes of the depression community (SDS10, SDS13, and SDS15) and SAS18 (edge weight: 0.02) of the anxiety community, among which the correlation with “Fatigue” (SDS10) was the strongest (edge weight: 0.03).

Not only was “Social contact or work affected by OC behavior” (YB7) positively correlated with SDS8 (edge weight: 0.04) of the depression community and five nodes of the anxiety community (SAS3, SAS6, SAS11, SAS16, and SAS18), among which the correlation with “Face flushing” (SAS18) was stronger (edge weight: 0.06), but it was also negatively correlated with “Emptiness” (SDS18) (edge weight: −0.01) of the depression community.

“Distress caused by OC behavior” (YB8) was positively correlated with SDS9 (edge weight: 0.02) of the depression community and four nodes of the anxiety community (SAS1, SAS2, SAS6 and SAS12), among which the correlation with “Tremors” (SAS6) was the strongest (edge weight: 0.07).

Not only was “Resistance to OC behavior” (YB9) positively correlated with two nodes of the depression community (SDS8 and SDS10) and SAS7 (edge weight: 0.001) of the anxiety community, among which the correlation with “Fatigue” (SDS10) was the strongest (edge weight: 0.04), but it was also negatively correlated with “Personal devaluation” (SDS17) (edge weight: −0.001) of the depression community.

“Control of OC behavior” (YB10) was not only positively correlated with SDS19 (edge weight: 0.08) of the depression community and seven nodes of the anxiety community (SAS1, SAS2, SAS3, SAS6, SAS11, SAS16, and SAS18), among which the correlation with “Face flushing” (SAS18) was the strongest (edge weight: 0.07), but also negatively correlated with three nodes of the depression community (SDS11, SDS12, and SDS18) and SAS13 (edge weight: −0.05) of the anxiety community, among which the correlation with “Psychomotor retardation” (SDS12) was the strongest (edge weight: −0.02). More detailed information on the correlations among nodes in the network is shown in **Supplementary Table S1**.

For the OC symptoms community, 26 edges ranged from 0.01 to 0.34, and the strongest correlation was between “Social contact or work affected by OC thinking” (YB2) and “Distress caused by OC thinking” (YB3). For the depression community, 86 edges ranged from −0.13 to 0.27, and the strongest correlation was between “Personal devaluation” (SDS17) and “Emptiness” (SDS18). For the anxiety community, 74 edges ranged from −0.06 to 0.30, and the strongest correlation was between “Weight loss” (SDS7) and “Confusion” (SDS11).

In the network of OC symptoms, anxiety, and depression in OCD, the 95% CI of edge weights was narrow, indicating that the accuracy of edge weights was acceptable (**Supplementary Figure S3-1**). The bootstrapped difference test for edge weights showed that the edge between “Control of OC behavior” and “Suicidal rumination” (YB10-SDS19) (edge weight: 0.08) and the edge between “Distress caused by OC behavior” and “Tremors” (YB8-SAS6) (edge weight: 0.07) were the largest edge weight between OC symptoms and depression and anxiety, respectively (**Supplementary Figure S3-2**).

**Figure 2:**
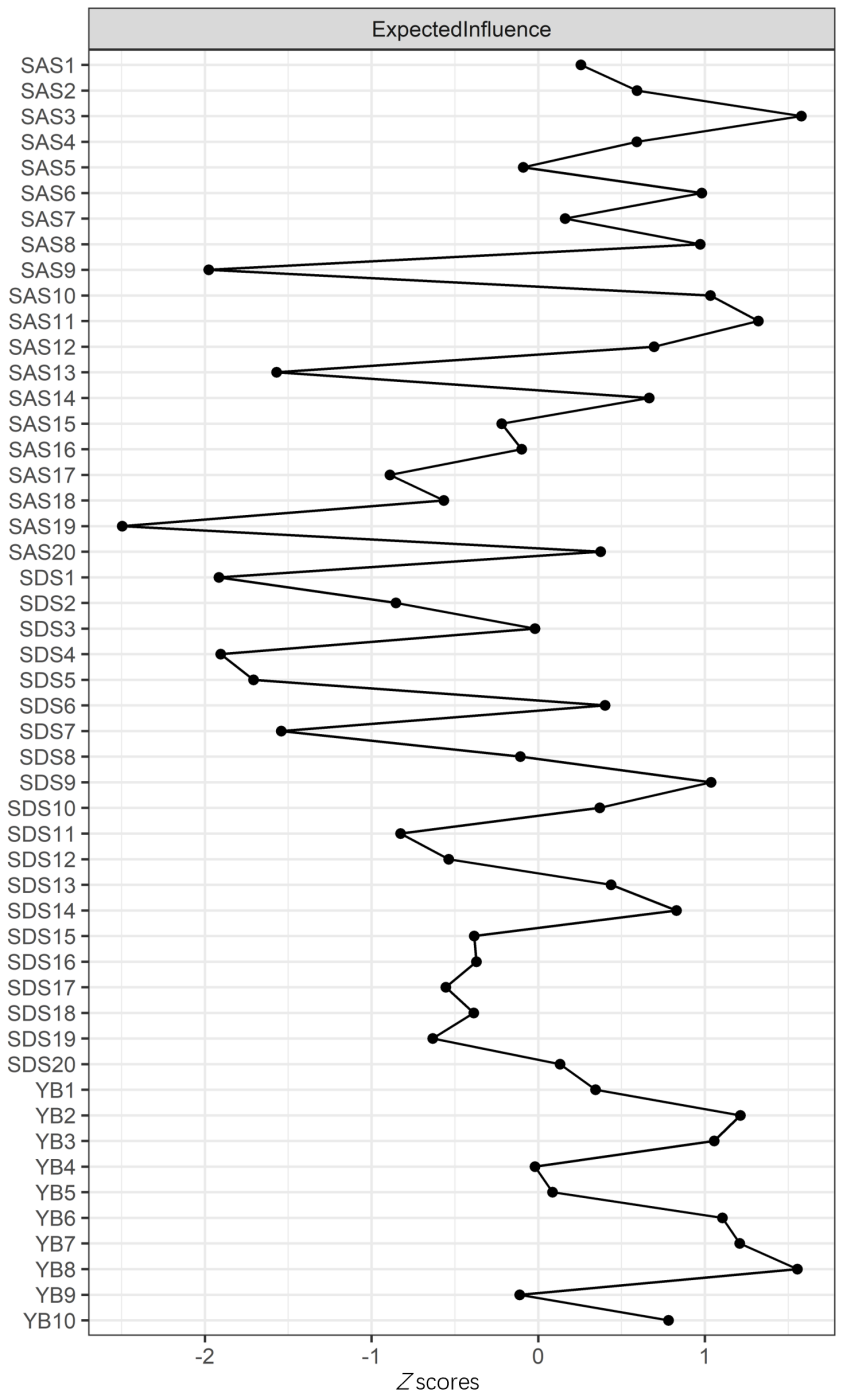
Centrality plot depicting the expected influence (EI) of symptoms. Higher EI values indicate greater importance in the network.

The node expected influence is shown in **Figure 2**. SAS3 “Panic” and YB8 “Distress caused by OC behavior” had the highest expected influence, indicating that the two variables were the most associated nodes in the network from a statistical viewpoint. SAS19 “Insomnia” had the lowest expected influence, indicating that this variable was the least associated node in the present network. As shown in **Supplementary Figure S3-3**, with the reduction of the subsample, the average correlation of the BEI indices of the original sample and the subsample decreased, while the CS coefficient of the node expected influence was 0.51, which was much larger than 0.25, indicating acceptable stability. Furthermore, bootstrapped difference tests for node expected influences showed that SAS3 and YB8, the highest expected influences, were significantly different to the other symptoms, at ratios of 65.31% and 69.39%, respectively (**Supplementary Figure S3-4**).

To assess the robustness of central and bridge symptoms, we re-estimated the network after excluding overlapping items (SDS10 and SAS8). The revised network retained “Panic” (SAS3) (EI: 1.23) and “Distress caused by OC behavior” (YB8) (EI: 1.18) as the top central symptoms. Bridge symptoms (e.g., SDS9 “Tachycardia” and SAS20 “Nightmares”) remained consistent, with 85% overlap in bridge nodes (Jaccard index: 0.72). Edge weights between communities were marginally reduced (mean Δ: 0.03) but statistically equivalent (95% CI: −0.05 to 0.11).

### 3.3 Bridge symptoms of OC symptoms, anxiety, and depression in individuals with OCD

**Figure 3:**
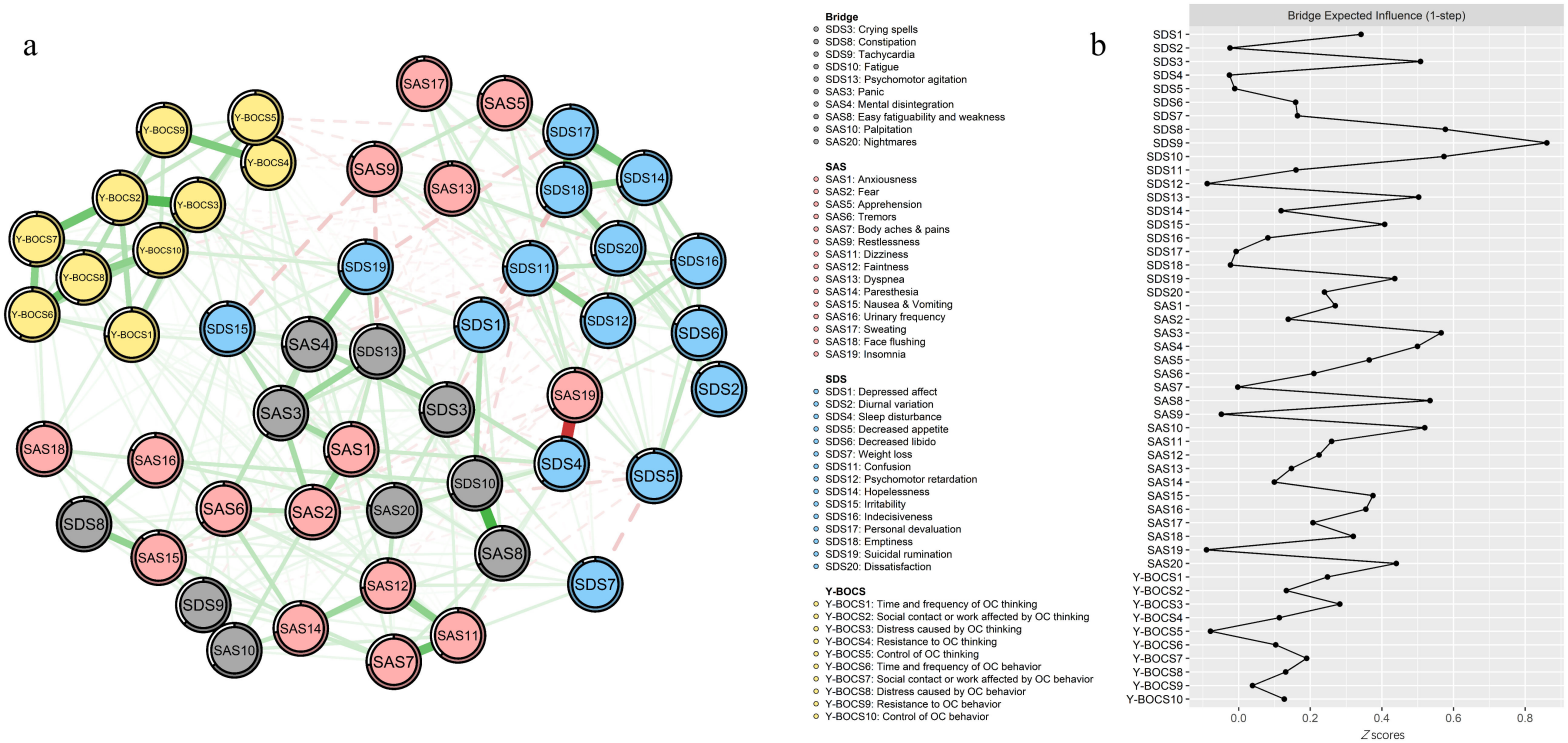
Network structure of OC symptoms, anxiety, and depression showing bridge symptoms. **(a)** Green edges represent positive correlations; red edges represent negative correlations. The thickness of the edge reflects the magnitude of the correlation. The circles around nodes depict their predictability. **(b)** Centrality plot depicting the BEI of OC symptoms, anxiety, and depression.

As shown in **Figure 3b**, SDS9 “Tachycardia” had the highest BEI, while SDS8 “Constipation,” SDS10 “Fatigue,” SDS3 “Crying spells,” and SDS13 “Psychomotor agitation” had a higher BEI in the depression community. Meanwhile, SAS3 “Panic” had the highest BEI, while SAS8 “Easy fatiguability, weakness,” SAS10 “Palpitation,” SAS4 “Mental disintegration,” and SAS20 “Nightmares” had a higher BEI in the anxiety community. These bridge symptoms were labeled with gray color in **Figure 3a**. The results indicated that the bridge nodes mentioned had the strongest connection with OC symptoms.

## 4 Discussion

In the study, we explored the network structure of OC symptoms, anxiety, and depression in 356 individuals with OCD using network analysis, aiming to elucidate the complex interplay among OC symptoms, anxiety, and depression and identifying potential pathways for clinical intervention. We found that the strongest edges exist within each disorder, which is in line with other network research focusing on the comorbidity of anxiety and depression (47, 50–54). The findings highlight 2 key patterns: (1) anxiety and depression communities exhibited stronger mutual connections compared to their associations with OCD symptoms, and (2) somatic, psychological, and sleep-related symptoms emerged as pivotal bridges linking OCD to anxiety/depression.

Among all the symptoms, the anxiety symptom “Panic” (SAS3) had the highest expected influence centrality, followed by the OC symptom “Distress caused by OC behavior” (YB8) and the anxiety symptom “Dizziness” (SAS11). The high expected influence centrality indicated that alleviating these nodes can destabilize the psychopathological networks and confer the highest general benefit to ease mental distress (24). That is to say, some interventions could be implemented to better alleviate OC symptoms, anxiety, and depression. For instance, “Panic” (SAS3) was identified as the core symptom of OCD accompanied by anxiety and depression, which is consistent with a previous study (55). According to the review, OCD and PD are notable but vary considerably, with rates from epidemiological and clinical studies ranging from 1.8% to 22% (56). Recently, the significance of co-occurring OCD and PD levels has been brought to the forefront clinically. From the perspective of etiology, as for biological contributors, general biological vulnerability factors consist of both genetic factors and trait neuroticism, which has been shown to predict the onset of both OCD and PD (57). Meanwhile, neuroticism as a trait has been an independent target for the genetic research of anxiety and related disorders, which has been found to be approximately 40% heritable (58). As for environmental contributors, stressful and traumatic life events during childhood (59–61), trauma (62, 63), a lack of parental care (64), and authoritarian (65) parenting have had an impact on co-occurring OCD/PD. From the perspective of psychological contributors, psychological vulnerabilities commonly elevated across both disorders, such as anxiety sensitivity or intolerance of uncertainty, may be the core factor of onset and development of co-occurring OCD/PD (66). Generalized psychological vulnerabilities refer to an enduring sense of uncontrollability for stressful life events, while specific psychological vulnerabilities describe the process that a person’s anxiety is focused on a particular concern (67). However, present studies on co-occurring OCD/PD are mainly based on the current empirical research, and further research of this overlap needs to be carried out. Our study provided evidence for the comorbidity between OCD and PD from the perspective of psychopathological network. Regarding “Distress caused by OC behavior” (YB8) in OC symptoms, it reflects a phenomenon that OCD may pay more attention to the distress caused by the OC behavior, and the distress further leads to the onset and development of other anxiety and depression symptoms. Perhaps, it can have a positive effect when the pain caused by OC behaviors is more focused on during psychiatric treatment and psychological counseling. Current OCD treatment strategies include the combination of selective serotonin reuptake inhibitors (SSRIs) and cognitive behavioral therapy (CBT) and the similarly oriented exposure and response prevention (ERP) method (68). Thus, it can be seen that the treatment of OCD based on OC behaviors has been put into practice. As for the anxiety symptom “Dizziness” (SAS11), there is a paucity of research on the relationship between single dizziness and OCD. Not only is dizziness one of the usual somatic symptoms (69), but it also occurs as a common adverse event with SSRIs (70). In this study, we did not control the first or second visit of individuals with OCD; thus, the co-occurring relationship between dizziness and OCD could not be inferred, which can be further studied.

Additionally, the results also revealed 10 bridge symptoms: “Tachycardia” (SDS9), “Constipation” (SDS8), “Fatigue” (SDS10), “Crying spells” (SDS3), “Psychomotor agitation” (SDS13), “Panic” (SAS3), “Easy fatiguability, weakness” (SAS8), “Palpitation” (SAS10), “Mental disintegration” (SAS4), and “Nightmares” (SAS20). Notably, somatic symptoms (“Tachycardia,” “Constipation,” “Fatigue,” “Easy fatiguability, weakness,” and “Palpitation”) accounted for the greatest proportion among the bridge symptoms, and “Tachycardia” (SDS9) had the highest BEI, indicating that it had the greatest liability for increasing the risk of comorbidity among OCD, anxiety, and depression, and thus deserved the utmost attention. The identification of somatic symptoms (e.g., tachycardia and palpitations) as bridges aligns with the somatic amplification theory, wherein heightened interoceptive awareness in OCD amplifies physiological arousal, perpetuating anxiety, and depressive symptoms (69). For instance, tachycardia may arise from autonomic hyperactivity during compulsive rituals, which individuals misinterpret as threatening, fueling anxiety (panic) and feelings of helplessness (depression). Similarly, constipation and fatigue may stem from chronic stress-induced gastrointestinal dysregulation and energy depletion, common in OCD. These somatic symptoms likely serve as shared biomarkers of physiological dysregulation across disorders (62). Moreover, psychological symptoms (“Psychomotor agitation” and “Mental disintegration”) also had higher BEI. The prevalence of comorbid OCD and psychosis showed significant variation ranging from 1.7% (71) to 14% (72). Psychological symptoms like psychomotor agitation and mental disintegration may reflect overlapping cognitive deficits. Impaired inhibitory control in OCD exacerbates rumination, which intersects with depressive cognitive styles (e.g., hopelessness) and anxious hypervigilance (73). It has been shown that comorbid OCD and psychosis were related to greater neuropsychological impairment and worse treatment outcome, with “Crying spells” (SDS3) appearing, as a result of emotional breakdown caused by psychological symptoms. Furthermore, “Nightmares” (SAS20), as an important indicator of sleep quality, was related to many psychiatric and psychological mental disorders. Nightmares, a sleep-related bridge, could arise from disrupted fear extinction processes, linking OCD-related intrusive thoughts to anxiety/depression via maladaptive emotional memory consolidation (74). Environmental factors, such as childhood trauma, may further entrench these connections by sensitizing neural circuits involved in threat detection (75). Improving sleep was closely associated with better mental health regardless of the severity and the comorbidity of mental health conditions (76), and it represents a potential treatment target (74, 77, 78).

Our findings indicated that the edge between “Control of OC behavior” (YB10) and “Suicidal rumination” (SDS19) as well as the edge between “Distress caused by OC behavior” (YB8) and “Tremors” (SAS6) were the largest edge weight between OC symptoms and depression and anxiety, respectively. The heightened connectivity between anxiety and depression aligns with their shared neurobiological substrates, such as dysregulation of the hypothalamic–pituitary–adrenal (HPA) axis and serotonergic pathways, which amplify negative affect and emotional hyperarousal (58, 67). In contrast, weaker OCD–anxiety/depression edges may reflect distinct cognitive–behavioral mechanisms. While OCD is characterized by threat overestimation and compulsive rituals to neutralize distress, anxiety and depression share broader negative emotionality and rumination (13, 66). This divergence underscores the need for targeted interventions addressing disorder-specific pathways alongside transdiagnostic bridge symptoms.

To some extent, our findings may enrich emerging research on how OCD occurs in the form of comorbidity with anxiety and depression and how to intervene better. Our network analysis of OCD, anxiety, and depression symptoms builds upon the previous work (73, 79), confirming the presence of distinct symptom clusters and the pivotal role of “bridge” symptoms in OCD-comorbid depression. Like these studies, we found “panic” and “distress caused by OC behavior” to be central to symptom interconnectivity, aligning with the consensus on their significance in OCD comorbidity. Unique to our study, the identification of somatic (e.g., “tachycardia” and “constipation”) and sleep-related (e.g., “nightmares”) bridge symptoms extend the understanding of the comorbidity network, suggesting novel targets for interventions that could alleviate the complex burden of these interrelated disorders. For example, biofeedback interventions to reduce tachycardia or cognitive restructuring to address somatic misinterpretations might alleviate panic and depressive affect. Similarly, improving sleep hygiene to mitigate nightmares could enhance emotional regulation across domains. These strategies align with the network theory’s premise that destabilizing central nodes cascades benefits system-wide (24).

Additionally, we have conducted gender-stratified analyses. In the male-only subgroup analysis, we found that the nodes representing “Panic” and “Distress caused by OC behavior” still had relatively high expected influence values, but the strength of some connections between OC symptoms and anxiety/depression symptoms differed compared to the overall sample. For example, the correlation between certain OCD-related behaviors and anxiety symptoms seemed to be stronger in men. In the female-only subgroup, the bridge symptoms also showed some differences. Somatic symptoms such as “Tachycardia” still had a high BEI, but the relationships between psychological symptoms and OCD symptoms were more complex compared to men. These results suggest that gender does have an impact on the network structure of OCD, anxiety, and depression symptoms.

There are some limitations in the present study. First and foremost, the network structure is specific to the scales we used in the study, which means that different assessment tools may form different network structures. Especially for the Y-BOCS, the scale is divided into two sections, obsessions and compulsions, whose connections of inner communities were much stronger than anxiety and depression communities. Secondly, longitudinal research is needed in the future. We cannot identify the directionality of the edges of communities and the causality among the nodes due to cross-sectional data. Thirdly, larger samples are needed. Fourthly, in terms of demographic information, participants in our study were mainly male because they were all recruited in the military hospital. Despite stratified analyses, gender imbalance and a wide age range may limit generalizability. For example, SAS8 (“Easy fatiguability”) and SDS10 (“Fatigue”) had overlapping roles in the full network (**Supplementary Table S4**), which persisted in subgroup analyses but needed further verification. Fifth, some scale items (e.g., SDS10 “Fatigue” and SAS8 “Easy fatiguability”) exhibit conceptual overlap, which may inflate cross-community correlations. Future research should validate these findings using scales with non-overlapping symptom domains.

## Data Availability

The raw data supporting the conclusions of this article will be made available by the authors, without undue reservation.
