# Supplementary figures and images for: The comorbidity of anxiety and depression symptoms in obsessive–compulsive disorder: a network analysis
Source: Front Psychiatry. 2025 May 2;16:1567448. doi: 10.3389/fpsyt.2025.1567448 (PMC12082660; doi:10.3389/fpsyt.2025.1567448)

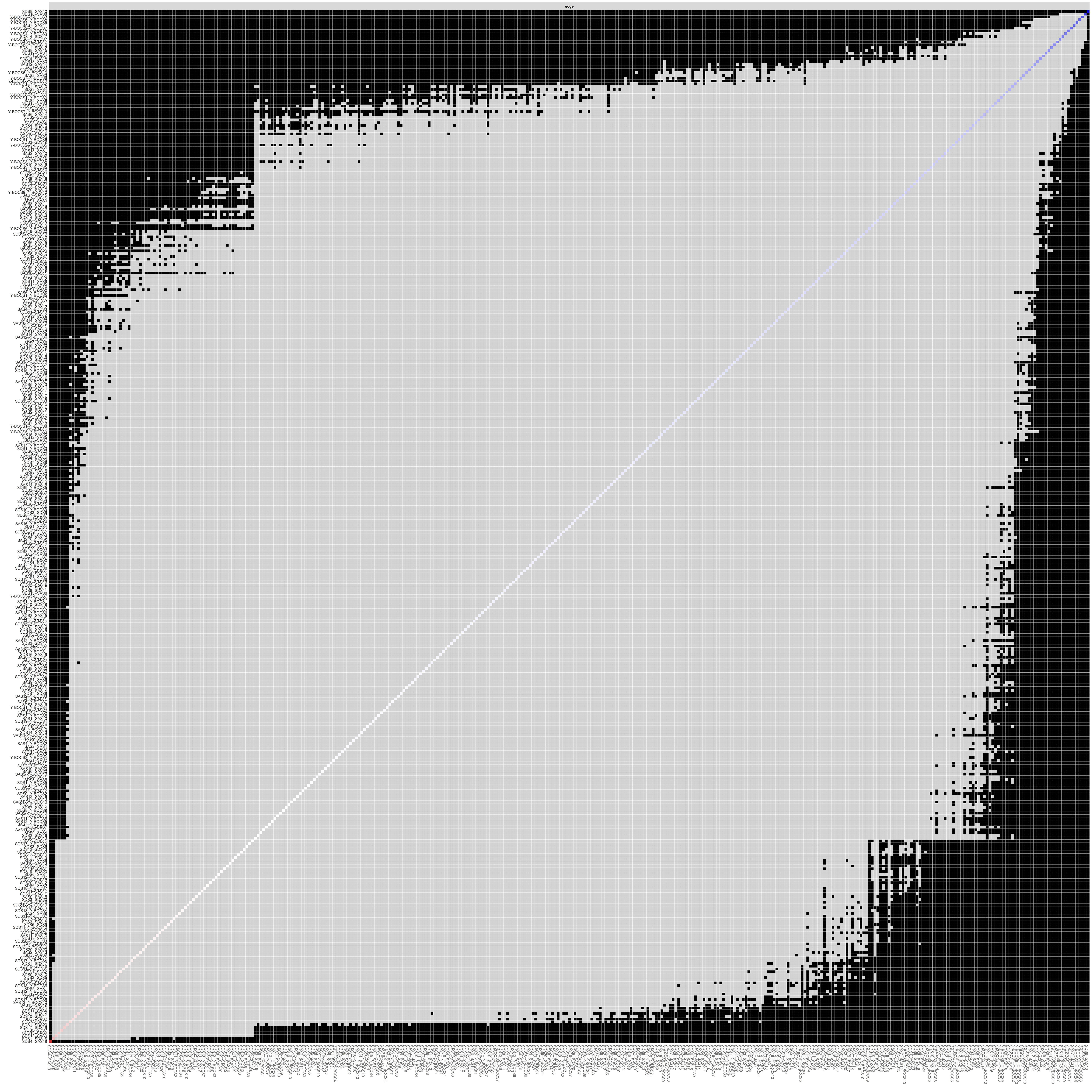

Supplement: Supplementary file 1 [file Image1.tiff]
