# Supplementary material for: The comorbidity of anxiety and depression symptoms in obsessive–compulsive disorder: a network analysis
Source: Front Psychiatry. 2025 May 2;16:1567448. doi: 10.3389/fpsyt.2025.1567448 (PMC12082660; doi:10.3389/fpsyt.2025.1567448)
Supplement: Supplementary file 3 [file Table1.docx]

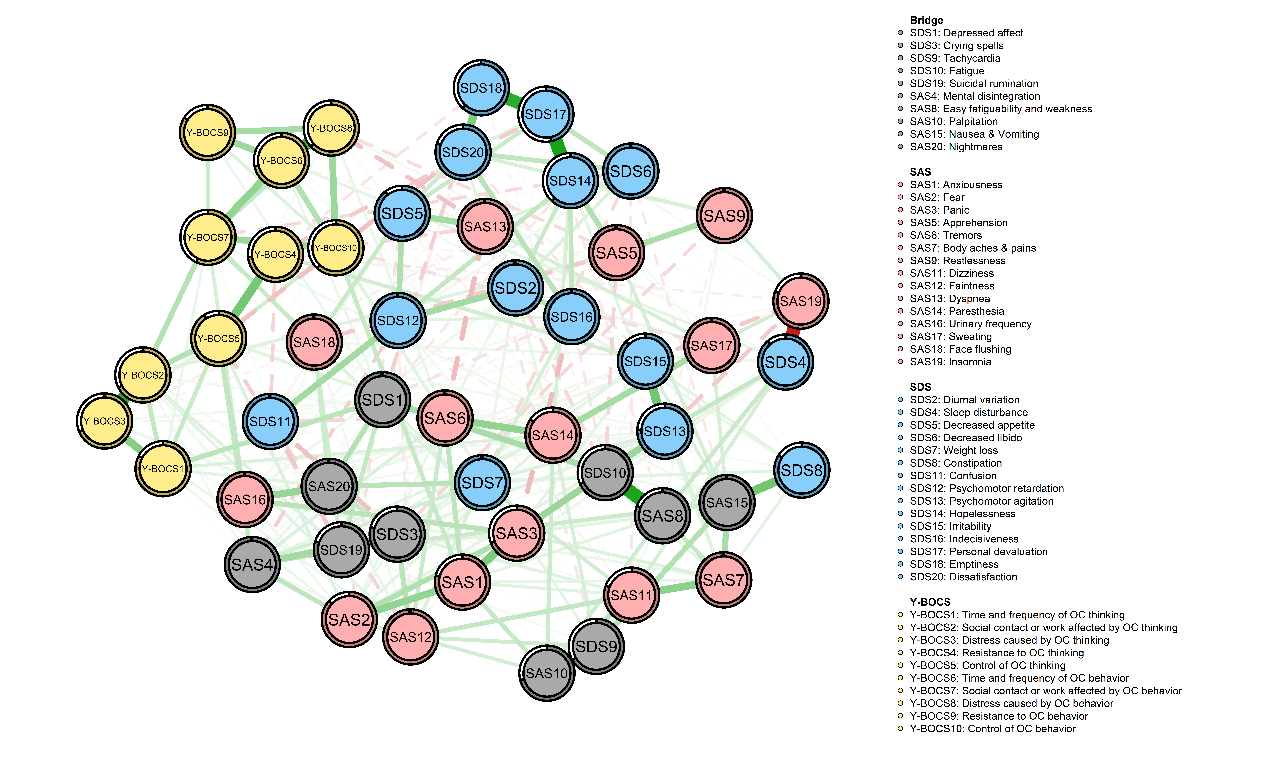


Fig.S1-1 Network structure of OCD, anxiety, and depression symptoms in female. Green edges represent positive partial correlations; red edges represent negative partial correlations. Edge thickness corresponds to correlation strength. Node predictability is indicated by the surrounding circles. Nodes are color-coded by symptom domain: red (SAS), blue (SDS), and yellow (Y-BOCS).


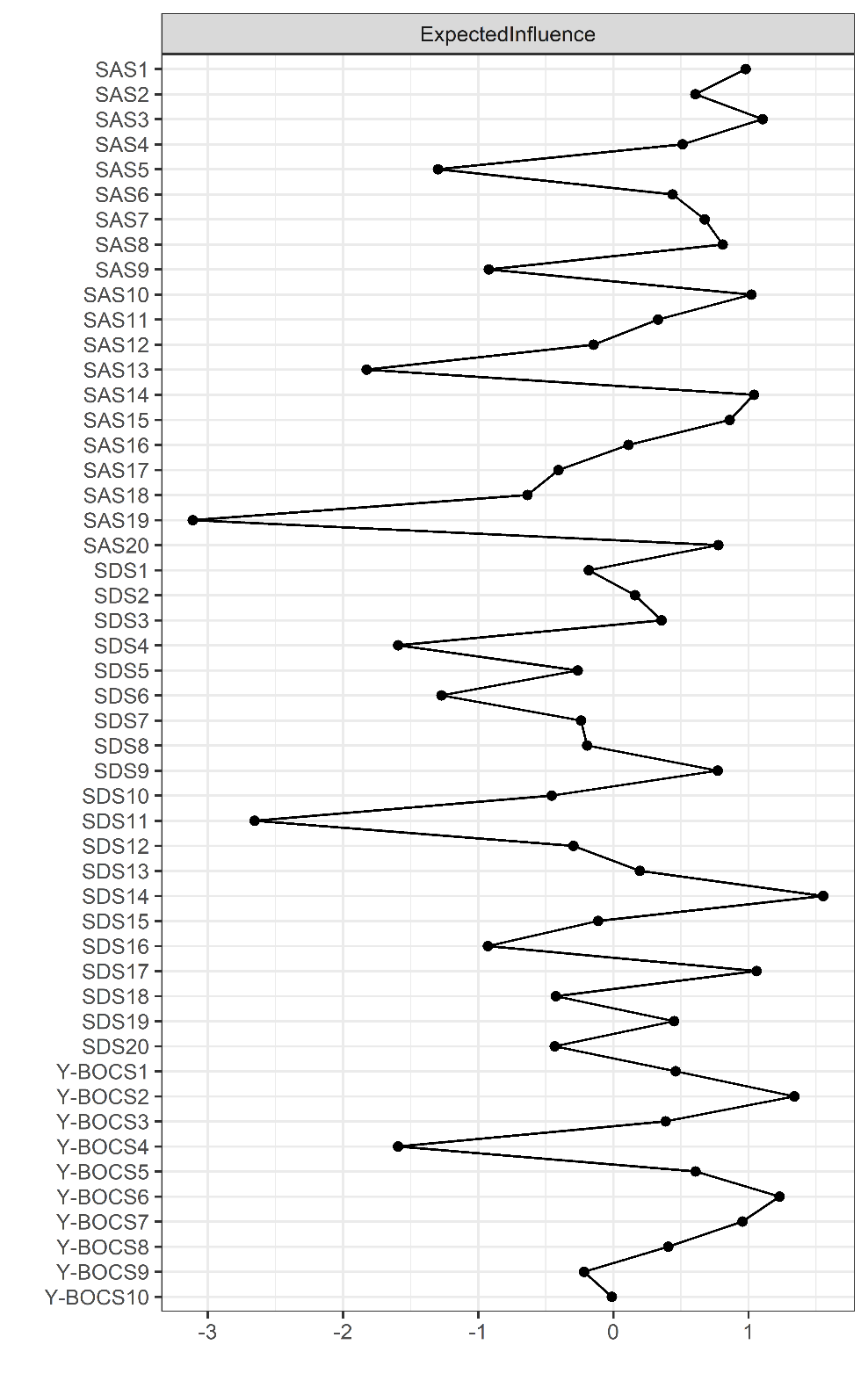


Fig.S1-2. Centrality plot depicting the expected influence (EI) of symptoms in female. Higher EI values indicate greater importance in the network.


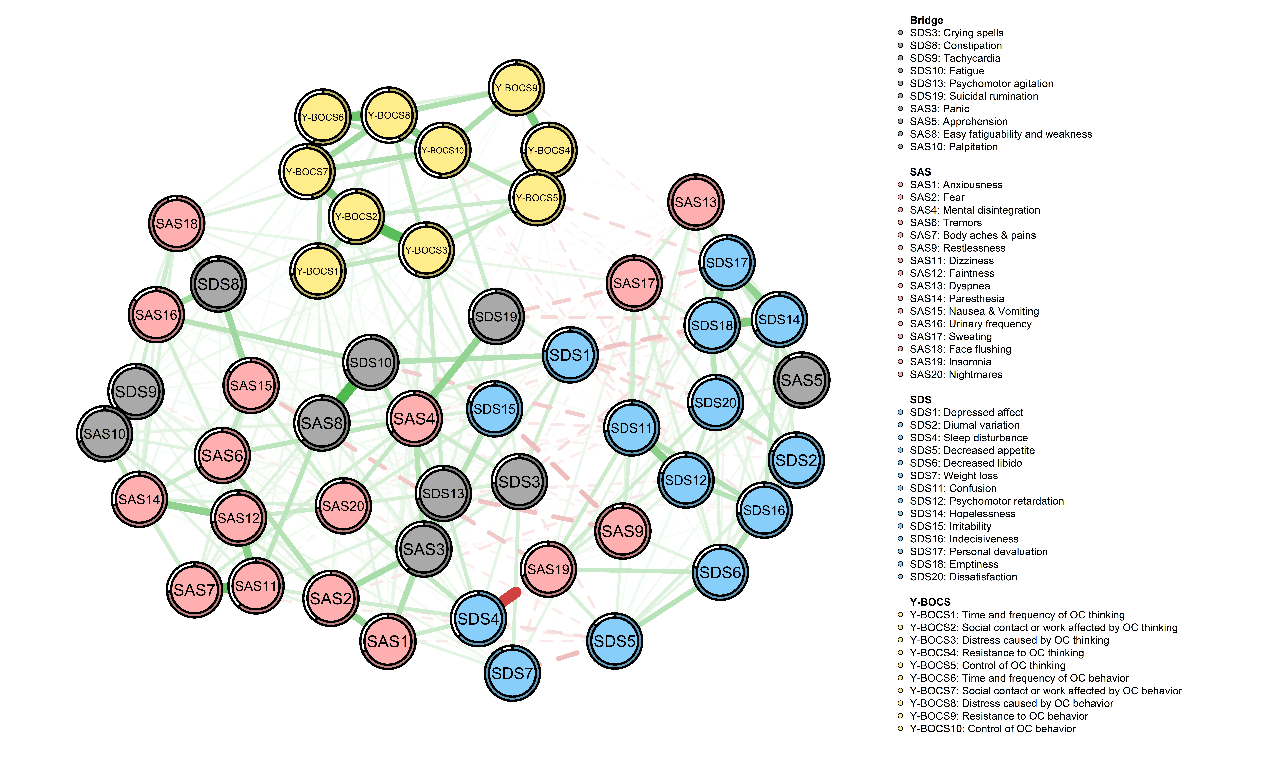


Fig.S1-3 Network structure of OCD, anxiety, and depression symptoms in male. Green edges represent positive partial correlations; red edges represent negative partial correlations. Edge thickness corresponds to correlation strength. Node predictability is indicated by the surrounding circles. Nodes are color-coded by symptom domain: red (SAS), blue (SDS), and yellow (Y-BOCS).


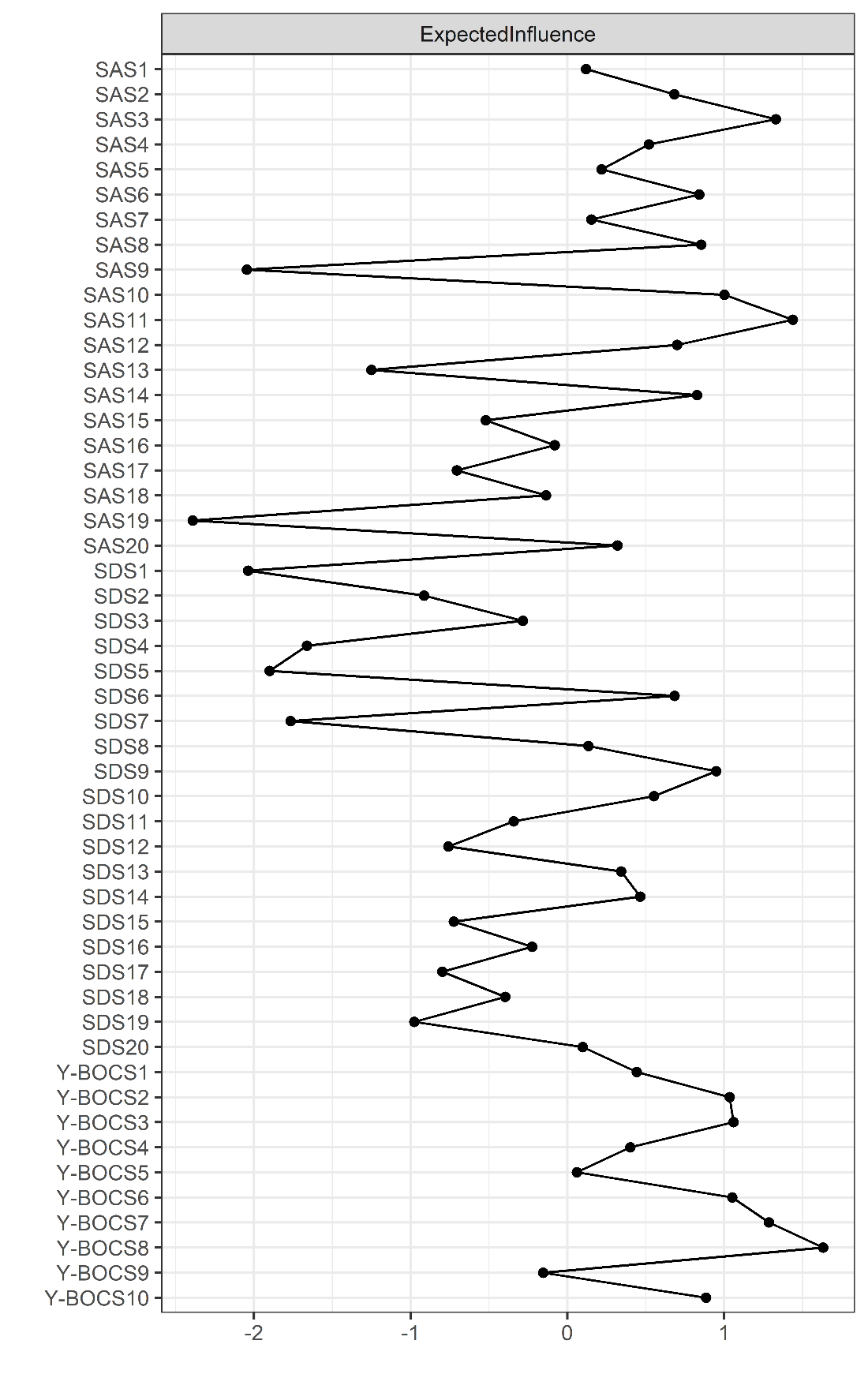


Fig.S1-4. Centrality plot depicting the expected influence (EI) of symptoms in male. Higher EI values indicate greater importance in the network.
