# Supplementary material for: The comorbidity of anxiety and depression symptoms in obsessive–compulsive disorder: a network analysis
Source: Front Psychiatry. 2025 May 2;16:1567448. doi: 10.3389/fpsyt.2025.1567448 (PMC12082660; doi:10.3389/fpsyt.2025.1567448)
Supplement: Supplementary file 5 [file Table3.docx]

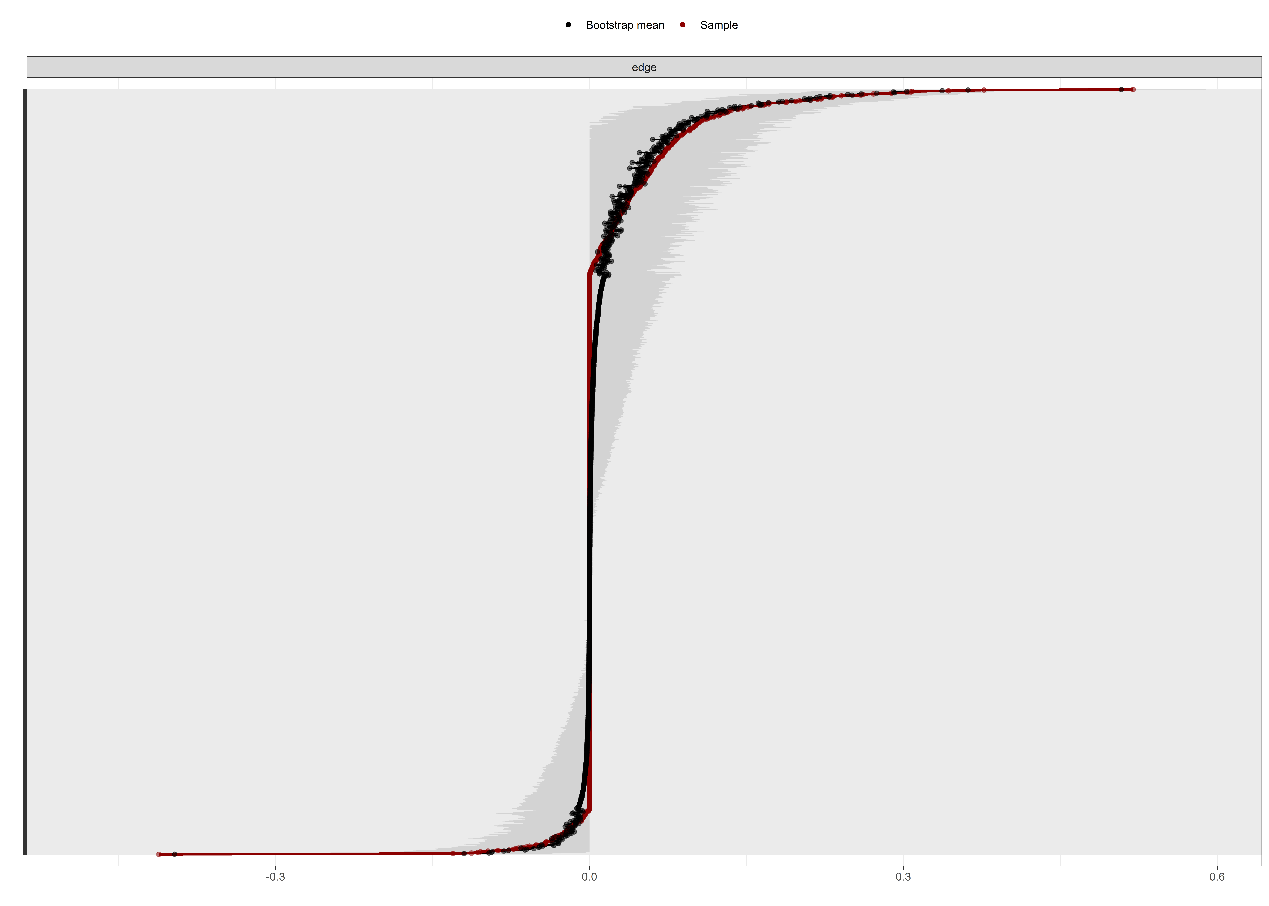


Figure S3-1. Accuracy of edge weights

*Note*: The red line depicts the sample edge weights and the gray bar depicts the bootstrapped confidence interval.


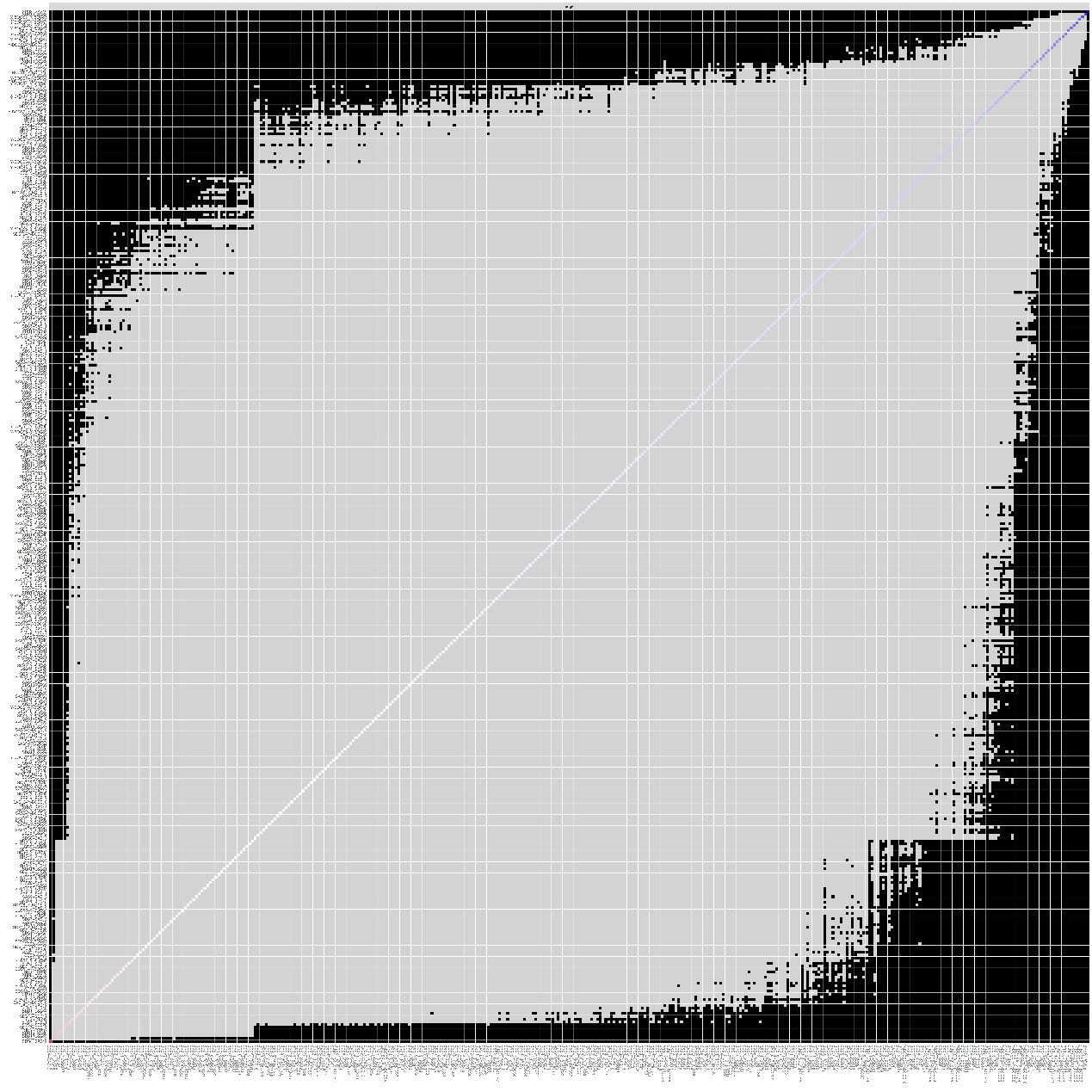


Figure S3-2. Bootstrapped difference test for edge weights

*Note*: Gray boxes indicate edge weights that do not differ significantly from one another, while black boxes indicate edge weights that do differ significantly. Blue and red boxes on the diagonal correspond to edge weights with positive and negative correlations, respectively.


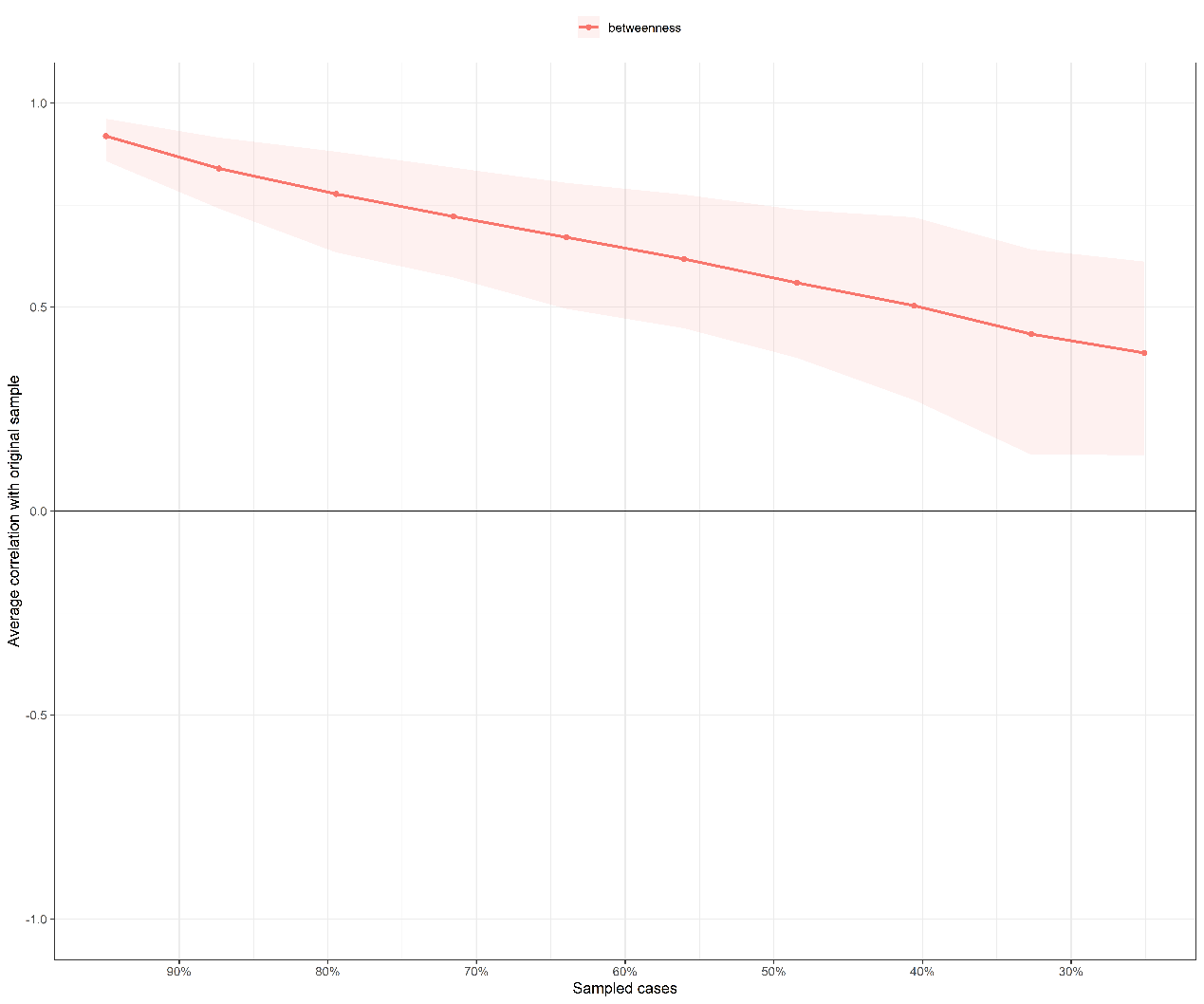


Figure S3-3. Stability of node bridge expected influences


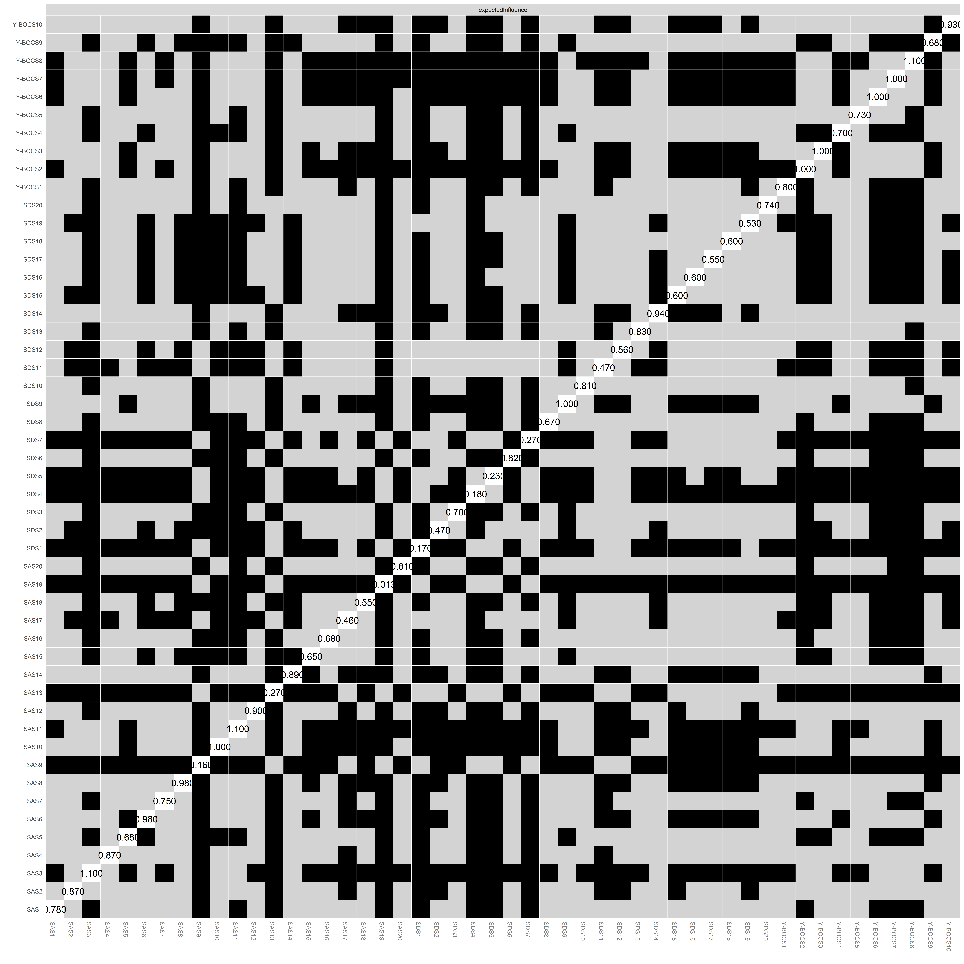


Figure S3-4. Bootstrapped difference test for node bridge expected influences

*Note*: Gray boxes indicate node bridge expected influences that do not differ significantly from one another, while black boxes indicate node bridge expected influences that do differ significant
